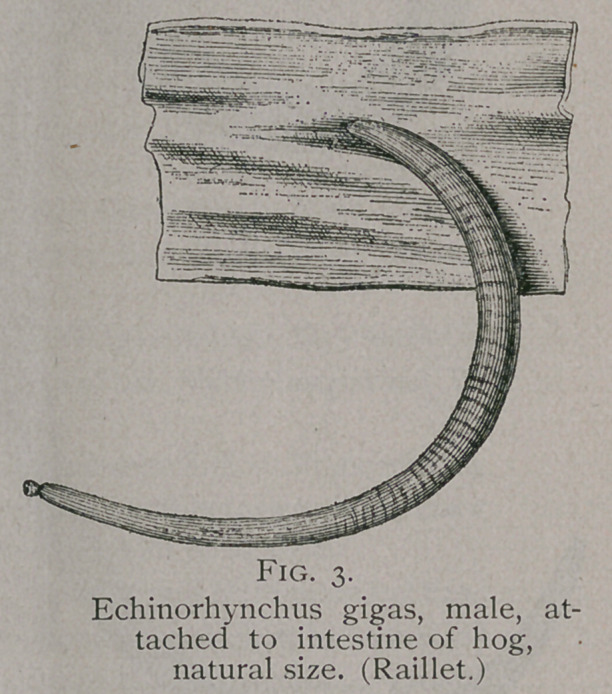# Echinorhynchus Gigas and Its Intermediate Host

**Published:** 1891-12

**Authors:** C. W. Stiles

**Affiliations:** Bureau of Animal Industry, Agricultural Department, Division of Pathology, Washington, D. C.


THE JOURNAL
OF
COMPARATIVE MEDICINE AND
VETERINARY ARCHIVES.

VOL. XII. DECEMBER, 1891. NO. 12.

### ECHINORHYNCHUS GIGAS AND ITS INTERMEDIATE HOST.

BY C. W. STILES, PH. D.

Bureau of Animal Industry,
Agricultural Department,
Division of Pathology,
Washington, D. C.

FIG. 1.—Echinorhynchus gigas, female, natural size. (Raillet.)

Among the parasites of the hog is found one species of the genus *Echinorhynchus* —*E. gigas.* In its adult stage this worm inhabits the small, rarely the large, intestines, the rostellum (its anterior extremity) buried in the intestinal wall, its body floating in the lumen. The presence of a large number of these parasites undoubtedly causes more or less irritation in the digestive tract of the host, and according to many authors it is not rare that one or more specimens bore through the wall into the abdominal cavity, thus causing peritonitis. Frequently *E. gigas* is found associated with Ascaris lumbricoides in the same animal, and although the two helminths are quite distinct, certain superficial observers have described *E. gigas* as *Asc. lumbricoides.* The following table will aid in determining the two species, so common in pigs :

| | Echinorhynchus Gigas. | Ascaris Lumbricoides. |
|---|---|---|
| Male, length | 6. 5—9 cm. | 15—25 cm. |
| Female, length | 20.—50 (ave. 35) cm. | 20—40 cm. |
| Color, | white. | yellowish. |
| Anterior extremity, | with armed rostellum, 6 rows of 8 hooks each. | with three lips. |
| Intestine, | totally wanting. | present. |
| Female genital opening, | posterior extremity. | on boundary between first and second thirds of the body. |
| Male genital opening, | posterior extremity. | near end of tail. |
| Eggs, | elongated, almost cylindrical, 0.087--0.1 mm. long, smooth, containing hooked embryo. | ovoid, 0.05—0.06 mm. long, rugged, generally without embryo when found in the fæces. |
| Development, | indirect with change of host. | direct without intermediate host. |

FIG. 2.

Cephalic extremity of Echinorhynchus gigas, enlarged ten diameters. (Raillet.)

FIG. 3.

Echinorhynchus gigas, male, attached to intestine of hog, natural size. (Raillet.)

Further, Ascaris has a very peculiar odor which is not noticed in Echinorhynchus. A microscopical examination of the fæces of the hog, is the only sure method of determining the presence of Echinorhynchus. If the parasites are present their eggs will be found and easily identified.

After this short introduction as to the nature of the worm, I will give a brief review of some experiments conducted in the Bureau of Animal Industry to determine the source from which the hogs become infected. For further particulars in regard to the anatomy, etc., of the parasite, see Kaiser's Monograph on *Echinorhynchus* in R. Leuckart's *Bibliotheca Zoologica*, 1890.

In 1868 Schneider stated that Melolontha vulgaris acted as secondary host for this helminth. Later (1887) Kaiser demonstrated that Cetonia aurata was also able to act as secondary host, and he believes further that C. aurata forms the regular source of infection of this curious and dangerous parasite.

As neither of these insects are found in the United States, the work of Schneider and Kaiser, both of Germany, fails, of course, to explain how the American swine become infected with the parasite in question, which occurs in nearly all sections of the country.

Noticing that the hogs around Washington, D. C., very commonly contain *E. gigas*, I determined to find the American insect in which the larval form of the parasite develops. As experiment animals I selected "white grubs" of the genus Lachnosterna and, placing a number of them in a flower pot, I gave them tender roots, etc., to eat, upon which I had sprinkled hundreds of eggs which I took from several female worms. The infection was made September 5th. On dissecting the insect larvæ, October 20th, I found them enormously infested with larvæ of Echinorhynchus in various stages of development. From one grub I took at least three hundred parasitic echinorhynchi. As I examined some of the grubs before the experiment, and found them free from the parasite and, as all the grubs examined later contained the characteristic larvæ, there seems to be no doubt that the experiment is positive.

Since making this infection I have learned from my friend, Mr. L. O. Howard, assistant entomologist of the Department, to whom I am indebted for the insects upon which I experimented, as well as for the entomological information in this paper, that it is the custom among many of our farmers to make use of their hogs in ridding their grounds of these grubs. If a portion of ground is found to be particularly infested with white grubs, the hogs are turned loose and destroy the grubs. This custom undoubtedly adds, to some extent, at least to the frequency of this parasite among American hogs; for, when a farmer feeds grubs to his hogs, he must necessarily feed them Echinorchynchi at the same time, provided the grubs are infected.

The white grub with which I have chiefly experimented, is the larva of *Lachnosterna arcuata.* According to Mr. Howard, this species has been reported from New York, New Jersey, District of Columbia, Georgia, Iowa and Missouri. The geographical distribution of the parasite in the United States is, however, very much greater than the distribution of this insect. It follows that *Lachnosterna arcuata* is not the only insect in America which can serve as the secondary host to our parasite. *L. dubia* has a much wider range than the former species, and Mr. Howard tells me that although it is practically impossible to distinguish the larval forms of the three species of Lachnosterna, I mention in this paper, he strongly mistrusts that I have both this and the following species among my experiment animals. Specimens of *L. dubia* are found in the National Museum, from Maine, Massachusetts, New York, New Jersey, District of Columbia, North Carolina, Ohio, Illinois, Wisconsin, Tennesee, Montana, Nevada, California and Texas. Another closely allied species, *L. hirticula* has been found in Massachusetts, New York, New Jersey, Pennsylvania, Maryland, District of Columbia, North Carolina, Illinois, Missouri, Nebraska and Minnesota.

These three species of insect are included in the old species *Lachnosterna fuseda*, Froehlich, and since they all have the same habits, feeding upon tender roots, etc., and each differ from one another only in their male genital organs, I assume provisionally that all three—in other words, Froehlich's *L. fusca*—can serve as secondary host for *E. gigas*, although I have given an absolutly positive demonstration only in the case of the first species, *L. arcuata.*

In all, ninety-one species of *Lachnosterna* are recognized in this country, and it seems to me highly probable that some of these other species may serve as secondary host for this parasite, although I have not as yet had an opportunity to experiment with them.

The typical group of the *Cetoniæ* is represented in this country by the genus *Euphoria*, of which we have sixteen species represented in all parts of this country, except in California. Of the closely allied genera *Allorhina* and *Gymnetis*, we have two species each found mainly in the Southern States. I hope later, through the kindness of Dr. D. E. Salmon, chief of this Bureau, to be able to extend my experiments to some of these other forms in order to determine whether only the *fusca* series of the *Lachnosterna* can serve as source of infection to our herds, or whether *E. gigas* can develop in other species of American insects as well.

Schneider's theory that *Melolontha vulgaris* is the secondary host of *E. gigas* in Europe, has met with some objection on the ground that this insect is essentially a phytophag, and not found in the dung heaps. Lachnosterna is also open to the same objection, but this objection it seems to me is only an apparent one, for the fæces of hogs are by no means confined to the dung heaps, but are found scattered over fields as well. Mr. Ashmead informs me that Lachnosterna grubs are found particularly frequent under the manure droppings in the fields, an occurence which is very satisfactorily explained by the fact that the roots of plants under the manure patches are particularly tender. Now it is perfectly evident that if the eggs of *E. gigas* are contained in the manure dropped upon the fields, they will, in course of time, be washed into the ground directly under the patch, and get upon the young roots of the plants. Upon eating these roots the insect larvæ can very easily become infected with the eggs of the parasite. Thus I see no objection to considering a phytophagous insect as a normal intermediate host for our parasite. While I thus support Schneider's Melolontha theory, I do not, of course, intend to detract any from the work of my friend Dr. Kaiser, to whom we are indebted for the finest monograph as yet published on the subject of Echinorhynchus.

## Figures and Tables

**Fig. 1. f1:**
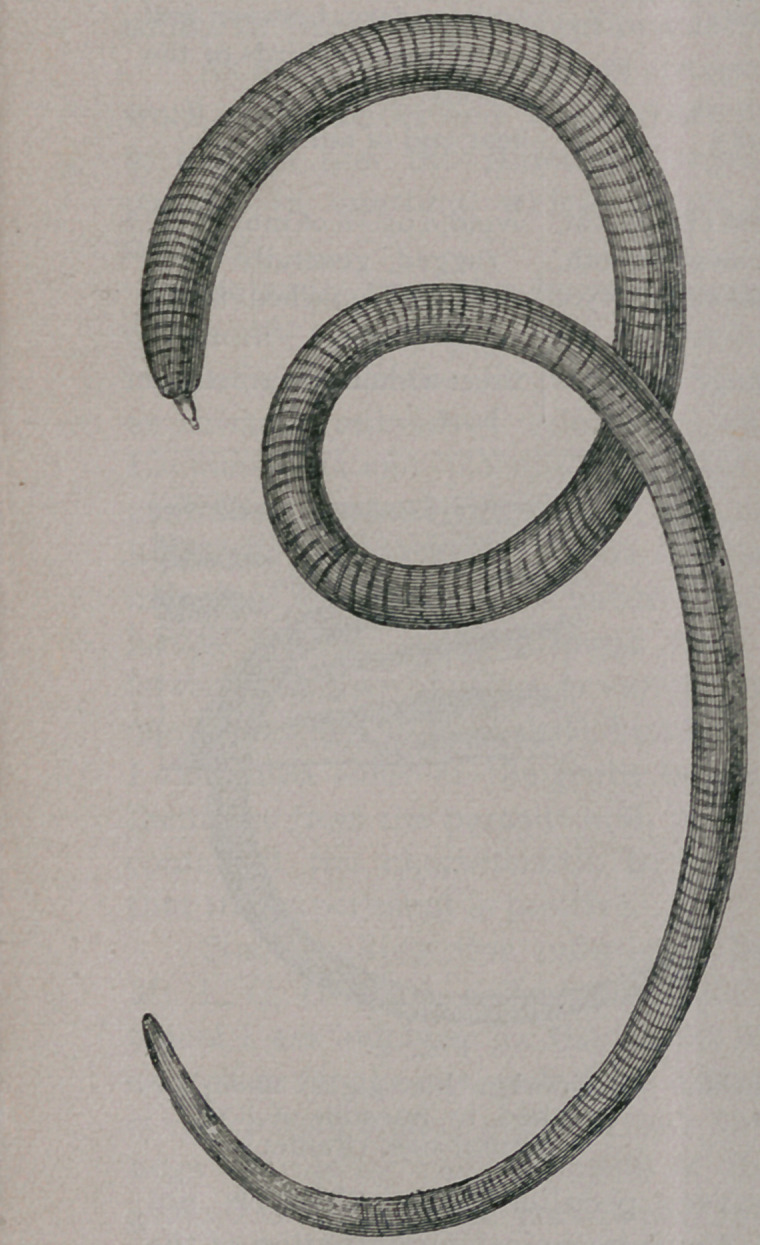


**Fig. 2. f2:**
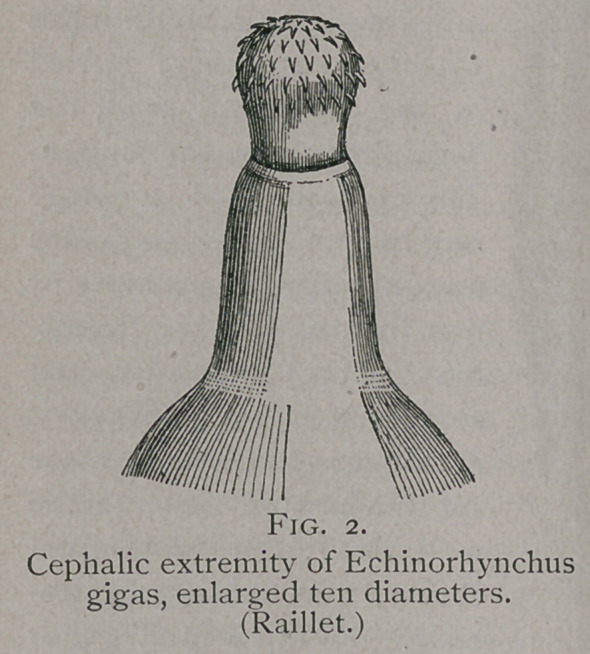


**Fig. 3. f3:**